# Dying from COVID-19 in nursing homes-sex differences in symptom occurrence

**DOI:** 10.1186/s12877-021-02228-4

**Published:** 2021-05-06

**Authors:** Lisa Martinsson, Peter Strang, Jonas Bergström, Staffan Lundström

**Affiliations:** 1 Department of Radiation Sciences, Umeå University, SE-90187 Umeå, Sweden; 2 Department of Oncology-Pathology, Karolinska Institutet, Stockholm, Sweden; 3 R & D department, Stockholms Sjukhem Foundation, Stockholm, Sweden; 4 Palliative Care Unit, Stockholms Sjukhem Foundation, Stockholm, Sweden

**Keywords:** COVID-19, Palliative care, End-of-life care, Nursing homes, Hospitals, Dementia

## Abstract

**Background:**

Coronavirus disease 2019 (COVID-19), is a disease with diverse presentation. Several studies have shown different occurrence of symptoms for women and men, but no studies have been found examining sex differences in clinical presentation for nursing home residents dying from COVID-19.

The objective of this study was to describe sex and age differences and the impact of a dementia diagnosis on symptom occurrence during the last week in life for persons dying from COVID-19 in nursing homes.

**Methods:**

This is a population-based retrospective study based on data from the Swedish Register of Palliative Care. A total of 1994 residents aged 65 or older who died from COVID-19 in nursing homes were identified. The impact of sex, age and a dementia diagnosis on six different symptoms was analysed using chi2-test and multivariate logistic regression.

**Results:**

Residents dying from COVID-19 were more often men (*p* < .002). Men more often had dyspnoea and death rattles (*p* < .001). Nausea was more common in women (p < .001). No sex differences in the occurrence of pain, anxiety or confusion were seen. Dyspnoea and nausea were less commonly reported in residents with dementia (*p* < .001).

**Conclusions:**

We found sex differences in symptom presentation for fatal COVID-19 in nursing home settings which remained after adjusting for age. Residents with a dementia diagnosis had fewer symptoms reported before death compared to those without dementia.

Clinical presentation of fatal COVID-19 differs between women and men in nursing homes. Residents with fatal COVID-19 present with more unspecific and less prominent symptoms when also suffering from dementia.

**Supplementary Information:**

The online version contains supplementary material available at 10.1186/s12877-021-02228-4.

## Introduction

Coronavirus disease 2019 (COVID-19), is a disease with diverse presentations. Common symptoms reported are fever, cough and dyspnoea [1]. A subset of COVID patients experience gastrointestinal symptoms [2]. Loss of taste or smell are reported among mild cases and dyspnoea is frequent among severe and fatal cases [1].

Men are more likely than women to die from COVID-19, and also more likely to be admitted to intensive care units due to COVID-19 [3]. Several studies suggest a sex difference in symptom presentation. Lechien et al. found that women with mild to moderate COVID-19 are slightly more prone to experience smell and taste dysfunction compared to men [4]. Biadsee et al. compared symptoms between women and men for a group of quarantined non-hospitalised patients and found that symptom presentation differs. Women were more likely to report a runny nose, anosmia and facial pain compared to men [5]. Pregnancy is also associated with a different clinical presentation [6].

Dementia is a known risk factor for death when infected by COVID-19 and is associated with an atypical presentation of symptoms [7]. Delirium as the first clinical presentation of COVID-19 was shown by Poloni et al. to be associated with old age and with multiple comorbidities, but not with sex, in a dementia facility [8]. In a Spanish study examining patients with COVID-19 admitted to an emergency department, older patients were less likely to present with fever, cough and dyspnoea, and more likely to present with unspecific symptoms such as confusion or presyncope/syncope [9].

In Sweden, around 82,000 people aged 65 and above live in nursing homes which corresponds to 0.8% of the total population. Swedish nursing homes are expected to deliver end-of-life care for those elderly patients who do not have complex palliative care needs. The quality of end-of-life care for persons dying from dementia in Sweden is better in nursing homes compared to hospitals [10]. Median time from admission to death is 24 months [11]. The most common underlying causes of death in this population are cardiovascular diseases and dementia, according to a Swedish register data study for deaths during 2011 and 2012 [12]. According to the Swedish National Board of Health and Welfare, 45% of all deaths from COVID-19 in Sweden up until the 2th November 2020 had occurred in nursing homes [13]. For many diagnoses, the occurrence of pain, dyspnoea, nausea, confusion, anxiety and death rattles is common during the last week in life [14]. The prevalence of these symptoms is measured during the last week in life by the Swedish Register of Palliative Care. A recent study from our group has shown significantly lower figures of dyspnoea for COVID-19 patients dying in nursing homes compared to those dying from COVID-19 in hospitals, 35% versus 73% [15]. Another extended study has shown that those nursing home residents who were acutely admitted and died in hospitals with COVID-19 had more breakthrough symptoms of breathlessness (60%) [16] i.e. a figure close to the 73% for hospital deaths in the first study. No studies have been found examining sex differences in clinical presentation for nursing home residents dying from COVID-19.

The objective of this study was to describe sex and age differences and the impact of a dementia diagnosis on symptom occurrence during the last week in life for persons dying from COVID-19 in nursing homes.

## Methods

Data were retrieved from the Swedish Register of Palliative Care (SRPC) [17–19]. Healthcare staff in Sweden answer an end-of-life questionnaire (ELQ) about the care given, focusing on the last week in life, after death of a patient. The registration is based on clinical knowledge and/or data from medical records. Large parts of the ELQ are only to be answered when a patient did not die unexpectedly, since the focus of the SRPC is to assess the quality of planned care given during the last week of life, irrespective of place of death, diagnosis or age. Around 60% of all deaths in the country are reported to the SRPC each year. The proportion of reported nursing homes deaths is around 75%.

The following inclusion criteria were used: age 65 or older, deceased during 2020, reported to the SRPC at the latest when data were retrieved, deceased in a nursing home (short-term or permanent stay). Symptom data from persons who died unexpectedly (i.e. the registrant answered “no” to the question “Based on the disease trajectory, was the death expected?”) are not registered in the ELQ and unexpected deaths were therefore excluded.

The following data were retrieved from the SRPC and used for analyses: occurrence and relief of six symptoms (dyspnoea, pain, nausea, anxiety, death rattles and confusion) during the last week in life, age, sex, diagnoses including COVID-19, the outcome of COVID-19 testing if done, whether death was expected, and days spent in nursing home before death, based on clinical knowledge and/or medical records data. Cases reported to have had an ongoing, suspected or previous COVID-19 infection were assigned to the COVID group, and cases not reported to have had COVID-19 were assigned to the non-COVID group.

Differences in mean age, sex distribution, comorbidities (heart disease, cancer, dementia), days in nursing home before death, and the occurrence of the six symptoms were analysed between those with COVID-19 and those without the disease using t-test and chi2-test. Symptom occurrence had been reported to the SRPC with items containing three different answers: “yes”, “no”, and “don’t know”, out of which the first two answers were included in the analysis.

The residents were divided into four age groups: 65–74, 75–84, 85–94 and 95 and older. Occurrence of the six symptoms were compared between women and men (chi2-test) and between the four age groups (logistic regression). A multivariate model was then used to examine the impact of age and sex in a combined model.

Occurrence of the six symptoms was also compared between those with and without dementia reported as a contributing cause of death (chi2-test). A multivariate model was then used to adjust the possible impact from dementia for age categories and sex.

Symptom relief was compared between women and men (chi2-test) and between the four age groups (logistic regression). The degree of symptom relief was dichotomised, comparing “completely” with the combined group of “partially” and “not at all”. A multivariate model was then used to examine the impact of age and sex in a combined model.

## Results

### Demographics and COVID-19 diagnosis

A total of 17,356 residents who had died in a nursing home in Sweden were reported to the SRPC during 2020 at the time of data collection. Of these, 17,118 were 65 years old or older,, out of whom 2203 were reported to have had COVID-19. Two hundred and nine persons reported to have had COVID-19 and 1122 persons not reported to have had COVID-19 died unexpectedly and were excluded. Ten persons were tested positive despite not being registered as having (or had) a COVID-19 infection and were excluded because of inconsistent information. After exclusion, 1994 remained in the COVID group and 13,783 in the non-COVID-group. In the COVID group, 1418 had an ongoing infection at time of death, 318 a suspected ongoing infection and 258 a previous infection. Out of these 1994 persons with COVID-19, 1632 (82%) were lab-verified with polymerase chain reaction (PCR) according to the healthcare staff reporting to the SRPC. Of the 13,783 in the non-COVID group, 2778 (20%) had been tested for COVID (Additional file 1: Appendix 1, Supplementary material).

There was a higher proportion of men in the COVID-19 group. The mean age was 87 (ranging from 65 to 107) in the COVID group and 87 (ranging from 65 to 108) in the non-COVID group and did not differ significantly between the groups. Heart disease and cancer were more common in the non-COVID group, while dementia was more common in the COVID group. Mean days spent in the nursing home before death did not differ. Dyspnoea, anxiety, death rattles and confusion were more common during the last week in life for the COVID group compared to the non-COVID group (Table 1).

**Table 1:** Comparison between the COVID and the non-COVID group reported from nursing homes to the SRPC during 2020

		COVID-19	Not COVID-19	*p* value
Age in years – mean (range)		87 (65–107)	87 (65–108)	0.31
Men – *n* (%)		858/1994 (43%)	5431/13783 (39%)	.002
Comorbidities (one person can have multiple comorbidities reported) – *n* (%)	Heart disease	563/1994 (28%)	4778/13783 (35%)	< .001
	Dementia	974/1994 (49%)	6269/13783 (46%)	.005
	Cancer	143/1994 (7%)	2546/13783 (19%)	< .001
Days in nursing home before death	Mean (range)	850 (0–6910)	858 (0–31,986)	.76
	Median	603	547	–
Dyspnoea		583/1892 (31%)	2309/13293 (17%)	< .001
Pain		1350/1938 (70%)	9668/13471 (72%)	.05
Nausea		176/1831 (10%)	1405/12800 (11%)	.08
Anxiety		1048/1873 (56%)	6800/12916 (53%)	.007
Death rattles		1005/1954 (51%)	6273/13573 (46%)	< .001
Confusion		433/1741 (25%)	2785/12385 (23%)	.026

Only expected deaths, based on the disease trajectory, were included. Comparison of occurrence of six symptoms during the last week in life between the COVID and non-COVID groups. Analysed with t-test and chi2-test

None of the six symptoms changed more than 5 percentage units in occurrence between 2019 and 2020 in the SRPC data.

### Sex and age differences in symptom occurrence in the COVID-19 group

In the COVID-19 group, the following occurrences of symptoms were reported: 31% dyspnoea, 70% pain, 10% nausea, 56% anxiety, 51% death rattles and 25% confusion. The proportion of residents having pro re nata (PRN) drugs against symptoms prescribed were high: 96% had an injection of opioids against pain, 91% had an injection against nausea, 95% had an injection against anxiety prescribed, and 95% had an injection against death rattles prescribed. In the COVID-19 group, dyspnoea and death rattles were more frequently reported in men, while nausea was more common in women (Table 2). When comparing age groups without adjusting for gender, dyspnoea was more often seen in the age group of 75–84 years compared to the oldest age category. No other differences in symptom occurrences were found between the different age groups (Additional file 1: Appendix 2, Supplementary material).

**Table 2:** Sex differences in symptom occurrence in the COVID group, not adjusted for age

	Women	Men	*p* value
Dyspnoea	295/1088 (27%)	288/804 (36%)	< .001
Pain	766/1113 (69%)	584/825 (71%)	.35
Nausea	120/1048 (11%)	56/783 (7%)	.002
Anxiety	591/1077 (55%)	457/796 (57%)	.27
Death rattles	531/1119 (47%)	474/835 (57%)	< .001
Confusion	241/988 (24%)	192/753 (25%)	.60

Analysed with chi2-test

When analysing symptom occurrence with both sex and age categories as independent variables in a multivariate regression model, the sex differences for the three symptoms remained: dyspnoea and death rattles were more common in men and nausea was more common in women (Table 3).

**Table 3:** Sex differences (ref: women) in symptom occurrence in the COVID-19 group, adjusted for age categories (ref: oldest).

	Sex differences (ref: women)			Age categories (ref: oldest)			
	OR	95% CI	*p* value	Age group	OR	95% CI	*p* value
Dyspnoea	1.44	1.18–1.77	< .001	65–74	.92	.57–1.49	.10
				75–84	1.31	.04–1.83	.73
				85–94	1.02	.75–1.38	.11
				≥ 95	Ref		
Pain	1.08	.88–1.31	.48	65–74	1.13	.71–1.18	.82
				75–84	1.14	.82–1.58	.61
				85–94	1.04	.77–1.39	.43
				≥ 95	Ref		
Nausea	.57	.41–.81	.001	65–74	1.14	.48–2.74	.76
				75–84	1.66	.94–2.92	.08
				85–94	1.47	.87–2.48	.16
				≥ 95	Ref		
Anxiety	1.08	.90–1.31	.40	65–74	1.01	.65–1.58	.96
				75–84	1.20	.89–1.63	.24
				85–94	1.05	.80–1.39	.73
				≥ 95	Ref		
Death rattles	1.47	1.22–1.77	< .001	65–74	.87	.56–1.35	.55
				75–84	.99	.73–1.33	.92
				85–94	1.02	.77–1.34	.91
				≥ 95	Ref		
Confusion	1.10	.88–1.38	.3 9	65–74	.77	.44–1.32	.34
				75–84	.80	.55–1.15	.23
				85–94	.96	.69–1.33	.79
				≥ 95	Ref		

Analysed with logistic regression

### Dementia diagnosis in relation to symptoms

Dyspnoea and nausea were less likely to be reported if the patient had dementia reported as a contributing cause of death (both *p* < .001) (Additional file 1: Appendix 3, Supplementary material). These differences remained after adjusting for sex and age categories (Table 4). The differences between men (more likely to suffer from dyspnoea and death rattles) and women (more likely to suffer from nausea) remained after dementia/no dementia was brought into the multivariate logistic model (Table 4). Dyspnoea was more common in the age group of 75–84 years compared to the oldest when adjusting for gender and dementia (Table 4).

**Table 4:** Multivariate logistic regression model showing differences in symptom occurrence between the groups with and without dementia as a contributing cause of death (ref: no dementia) within the COVID group, adjusted for age categories (ref: oldest) and sex (ref: women)

	Dementia (ref: no dementia)			Sex differences (ref: women)			Age categories (ref: oldest)			
	OR	95% CI	*p* value	OR	95% CI	*p* value	Age group	OR	95% CI	*p* value
Dyspnoea	.64	.53–.78	< .001	1.42	1.15–1.73	.001	65–74	1.00	.61–1.63	.99
							75–84	1.45	1.04–2.04	.030
							85–94	1.09	.80–1.50	.57
							≥ 95	Ref		
Pain	.91	.75–1.11	.36	1.07	.87–1.31	.51	65–74	1.15	.72–1.85	.56
							75–84	1.16	.84–1.61	.37
							85–94	1.05	.78–1.41	.74
							≥ 95	Ref		
Nausea	.49	.35–.68	< .001	.55	.39–.78	.001	65–74	1.34	.55–3.22	.52
							75–84	1.92	1.08–3.39	.026
							85–94	1.64	.97–2.79	.07
							≥ 95	Ref		
Anxiety	.89	.74–1.07	.23	1.08	.89–1.30	.44	65–74	1.03	.66–1.61	.89
							75–84	1.23	.91–1.68	.19
							85–94	1.07	.81–1.42	.63
							≥ 95	Ref		
Death rattles	1.05	.88–1.26	.60	1.47	1.22–1.78	<.001	65–74	.87	.56–1.34	.52
							75–84	.98	.72–1.32	.87
							85–94	1.01	.76–1.33	.95
							≥ 95	Ref		
Confusion	1.05	.84–1.31	.67	1.11	.88–1.39	.38	65–74	.76	.44–1.31	.33
							75–84	.79	.55–1.14	.21
							85–94	.95	.68–1.33	.76
							≥ 95			

### Sex difference in symptom relief in the COVID-19 group

Out of the residents that were reported to have suffered from dyspnoea or death rattles, complete relief was obtained for more women than men (for dyspnoea: 51% compared to 44%, *p* = .049, for death rattles: 59% compared to 49%, *p* = .001), Additional file 1: Appendix 4, Supplementary material. For the other four symptoms, there were no sex differences regarding complete symptom relief (Additional file 1: Appendix 4, Supplementary material). No differences between the age categories regarding complete symptom relief were found (Additional file 1: Appendix 5, Supplementary material). When adjusting for age categories, the difference between men and women regarding complete relief of dyspnoea and death rattles remained (Table 5).

**Table 5:** Sex differences (ref: women) in complete symptom relief (complete vs partial or not at all) in the COVID-19 group for those reported to have suffered from that symptom during the last week, adjusted for age categories (ref: oldest).

	Sex differences (ref: women)			Age categories (ref: oldest)			
	OR	95% CI	*p* value	Age group	OR	95% CI	*p* value
Dyspnoea	.72	.52–.998	0.049	65–74	1.54	.68–3.49	.31
				75–84	1.15	.66–2.00	.63
				85–94	1.27	.75–2.16	.38
				≥ 95	Ref		
Pain	.94	.70–1.26	.68	65–74	.79	.39–1.60	.51
				75–84	.67	.40–1.11	.12
				85–94	.80	.50–1.28	.35
				≥ 95	Ref		
Nausea	.86	.45–1.66	.65	65–74	.39	.07–2.20	.29
				75–84	.43	.14–1.29	.13
				85–94	.82	.29–2.34	.71
				≥ 95	Ref		
Anxiety	.90	.67–1.20	.46	65–74	.71	.36–1.40	.33
				75–84	.96	.59–1.56	.88
				85–94	.93	.60–1.45	.76
				≥ 95	Ref		
Death rattles	.67	.52–.86	.002	65–74	1.13	.61–2.10	.70
				75–84	1.05	.69–1.60	.83
				85–94	1.09	.74–1.61	.67
				≥ 95	Ref		
Confusion	.89	.59–1.34	.57	65–74	1.65	.63–4.30	.31
				75–84	.67	.34–1.29	.23
				85–94	.88	.49–1.57	.66
				≥ 95	Ref		

Analysed with logistic regression

## Discussion

This study shows that there are sex differences in symptom presentation for fatal COVID-19 in nursing home settings which remain after adjusting for age. Men dying from COVID-19 in nursing homes more often suffered from dyspnoea and death rattles compared to women. Women more often suffered from nausea. These sex differences remained after adjusting for age. A hypothesis is that part of the difference regarding dyspnoea could be due to a higher incidence of lung diseases among men in this population. This could be caused by more smoking, a higher presence of chronic obstructive pulmonary disease (COPD) and more occupational lung injuries. In Sweden, women born before 1940 more seldom have had a period of smoking compared to men. For women born 1940 or later, it is more common to have had a period of smoking. Mortality from COPD is higher for men than for women, although mortality for women is expected to increase in the future [20]. Further research is needed to evaluate whether previous smoking and/or previous occupational injuries affect the symptom burden for fatal COVID-19.

Nausea was more common in female residents. It is known that a subset of COVID-19 patients experience gastrointestinal symptoms [2] and that women have these symptoms more often [4]. We have found no previous study reporting this in fatal cases. However, women have been reported to be more prone to experience nausea in other health care areas. E.g., women more often experience nausea as presenting symptom of acute coronary syndrome compared to men, for unknown reasons [21–23], and have a higher risk to experience chemotherapy-induced nausea [24].

In contrast to the findings by Martín-Sánchez et al. that older people exhibit less COVID-specific symptoms and more atypical symptoms [9], age was not a main factor explaining symptom occurrence in COVID-19 in our studied population. The residents had similar symptoms regardless of age, with the exception of dyspnoea being more common in ages 75–84 compared to younger and older. However, the study population consisted of nursing home residents with a median time from admission to death of 24 months [11]. This population differs from the elderly population as a whole and thus the results are not necessarily generalisable to a population of similar age not living in nursing homes. Old age has previously been shown to be associated with lower reported symptom burden in a geriatric population in need of palliative care [25]. Our group has previously shown that a subset of nursing home residents who died in hospitals with COVID-19 more often had breathlessness and delirium during the last week in life compared to nursing home residents with COVID-19 who died in their nursing home [16].

We do not have national data on days from diagnosis or onset of symptoms up to death, but it is known that the median number of days from symptom onset to death was 8 days based on 481 deaths in nursing homes in the Stockholm region during the spring 2020, which is much shorter than the average patient dying from COVID-19 in hospitals [16]. The proportion of nursing home residents who died related to COVID-19 in Sweden during 2020 out of all COVID-19-related deaths is similar to that reported from other countries [26]. Sweden is considered advance care planning-naïve on a national level without legally binding advance directives and a lack of support for advance care planning discussions. This also applies to nursing homes. As our group previously has reported, 74% of all persons who died expectedly according to their disease trajectory with COVID-19 in hospitals and nursing homes and were reported to the SRPC up until 19th May 2020 had had an end of life discussion [27].

For most of the study period (from April 1st to September 30th 2020), there were national restrictions on family visitations in the nursing homes. Also, individual care homes had visitation restrictions before and after that period. Nursing home residents receiving end-of-life care were usually allowed visits, but during the spring 2020 this was complicated by lack of personal protective equipment. Not being able to receive family visits might have affected both symptom detection and symptom experiences. It is also possible that the heavy burden on individual care homes during outbreaks made it more difficult to detect and alleviate symptoms for individuals infected during the outbreak.

This study gives further support to previous reports that dementia is associated with less clinically detected dyspnoea and more unspecific symptoms [7, 8]. However, no association was found between dementia and confusion/delirium as seen in other studies [8, 9]. It is not clear whether residents with dementia truly experience fewer symptoms or whether the differences are due to communication difficulties. However, the clinical implication is that nursing home residents with fatal COVID-19 present with more unspecific and less prominent symptoms when also suffering from dementia.

### Strengths and limitations

This is a large population-based register study using a pre-existing validated data collection method.

COVID-19 has been reported by the healthcare staff registering in the SRPC. Our data have not been verified via the Cause of Death Register at the National Board of Health and Welfare. Furthermore, mainly because of limited testing capacity during the first months of the pandemic, a substantial part of the COVID-19 cases had not been PRC verified but were reported to the SRPC by the healthcare staff based on the clinical presentation.

Information about functional status before death, detailed information about the dementia diagnosis, and around comorbidities other than the direct causes of death, would have enabled a better understanding of the sample as well as a better comparison between the groups. Unfortunately, these data are not collected by the register and implies limitations to the study.

A limitation is that the symptom data do neither contain information about who did the assessment (patient or staff) nor data on symptom severity, as the occurrence of symptoms are registered in a yes/no format. However, when a symptom is present, symptom relief is graded in three steps (complete, partial, no relief).

There were fewer than 10 cases in the youngest age group available for the analysis of complete symptom relief of nausea, and thus the symptom relief of nausea analysis was underpowered.

## Conclusions

There are sex differences in symptom presentation for fatal COVID-19 in nursing home settings which remain after adjusting for age. Moreover, residents with dementia have fewer and more unspecific symptoms reported before death compared to those without dementia.

## Supplementary Information

**Additional file 1: Appendix 1**. Number of persons with and without COVID-19 infection and outcome of COVID-19 testing. **Appendix 2**. Age differences in symptom occurrence in the COVID-19 group, not adjusted for sex. Analysed with logistic regression. **Appendix 3**. Differences in symptom occurrence between the groups with and without dementia as a contributing cause of death within the COVID-19 group, not adjusted for age or sex. Analysed with chi2-test. **Appendix 4**. Sex differences in complete symptom relief (complete vs partial or not at all) in the COVID-19 group for those reported to have suffered from that symptom during the last week, not adjusted for age. Analysed with chi2-test. **Appendix 5**. Age differences in complete symptom relief (complete vs partial or not at all) in the COVID-19 group for those reported to have suffered from that symptom during the last week of life, not adjusted for sex. Analysed with logistic regression

## Data Availability

The dataset contains personally identifying information, such as personal identity numbers, and potentially identifying information, such as date of death, and therefore is subject to ethical and legal restrictions on public sharing. We cannot share the dataset, which is on individual level, because it is not permitted according to the laws that apply in Sweden.

## Abbreviations

COPD: Chronic obstructive pulmonary disease
COVID-19: Coronavirus disease 2019
ELQ: End-of-life questionnaire
PRN: Pro re nata
SRPC: Swedish Register of Palliative Care
