# Supplementary material for: Dying from COVID-19 in nursing homes-sex differences in symptom occurrence
Source: BMC Geriatr. 2021 May 6;21:294. doi: 10.1186/s12877-021-02228-4 (PMC8100361; doi:10.1186/s12877-021-02228-4)
Supplement: Supplementary file 1 — Additional file 1: Appendix 1. Number of persons with and without COVID-19 infection and outcome of COVID-19 testing. Appendix 2. Age differences in symptom occurrence in the COVID-19 group, not adjusted for sex. Analysed with logistic regression. Appendix 3. Differences in symptom occurrence between the groups with and without dementia as a contributing cause of death within the COVID-19 group, not adjusted for age or sex. Analysed with chi2-test. Appendix 4. Sex differences in complete symptom relief (complete vs partial or not at all) in the COVID-19 group for those reported to have suffered from that symptom during the last week, not adjusted for age. Analysed with chi2-test. Appendix 5. Age differences in complete symptom relief (complete vs partial or not at all) in the COVID-19 group for those reported to have suffered from that symptom during the last week of life, not adjusted for sex. Analysed with logistic regression [file 12877_2021_2228_MOESM1_ESM.docx]

# Supplementary material: Dying from COVID-19 in nursing homes – sex differences in symptom occurrence

## Authors

Lisa Martinsson MD, PhD^a^
Peter Strang MD, PhD, Professor of Palliative Medicine^b, c^

Jonas Bergström, MD^d^

Staffan Lundström, MD, PhD, Associate Professor^b, c^

^a^ Department of Radiation Sciences, Umeå University, Umeå, Sweden

^b^ Department of Oncology-Pathology, Karolinska Institutet, Stockholm

^c^ R & D department, Stockholms Sjukhem Foundation, Stockholm, Sweden

^d^ Palliative Care Unit, Stockholms Sjukhem Foundation, Stockholm, Sweden

## Corresponding author

Lisa Martinsson MD, PhD, Department of Radiation Sciences, Umeå University, SE-90187 Umeå, Sweden
lisa.martinsson@umu.se

|  | PCR test for SARS-CoV-2 | | | | | Total |
| --- | --- | --- | --- | --- | --- | --- |
|  | Positive | Negative | Tested, no answer when reporting to the SRPC | Not tested | Unknown if tested/not reported if tested |  |
| Ongoing COVID-19 infection at death | 1401 | 10 | 0 | 5 | 2 | 1418 |
| Clinically suspected ongoing COVID-19 infection | 9 | 91 | 26 | 154 | 38 | 318 |
| Previous COVID-19 infection | 222 | 19 | 0 | 13 | 4 | 258 |
| COVID-19 not reported | 10* | 2739 | 39 | 3277 | 7728 | 13793 |
| Total | 1642 | 2859 | 65 | 3449 | 7772 | 15787 |

**Appendix 1.** Number of persons with and without COVID-19 infection and outcome of COVID-19 testing.
*Excluded from further analysis because of inconsistent information

|  | Age group | Symptom occurrence | OR | 95% CI | p value |
| --- | --- | --- | --- | --- | --- |
| Dyspnoea | 65–74 | 35/122 (29%) | 1.04 | .64–1.68 | .87 |
|  | 75–84 | 194/537 (36%) | 1.46 | 1.05–2.03 | .023 |
|  | 85–94 | 284/982 (29%) | 1.05 | .77–1.43 | .75 |
|  | ≥ 95 | 70/251 (28%) | Ref | | |
| Pain | 65–74 | 89/125 (71%) | 1.16 | .73–1.85 | .54 |
|  | 75–84 | 394/553 (71%) | 1.16 | .84–1.60 | .36 |
|  | 85–94 | 690/1000 (69%) | 1.04 | .78–1.40 | .78 |
|  | ≥ 95 | 177/260 (68%) | Ref | | |
| Nausea | 65–74 | 8/113 (7%) | .94 | .40–2.24 | .90 |
|  | 75–84 | 54/525 (10%) | 1.42 | .81–2.48 | .22 |
|  | 85–94 | 96/952 (10%) | 1.34 | .82–2.35 | .22 |
|  | ≥ 95 | 18/241 (8%) | Ref | | |
| Anxiety | 65–74 | 64/117 (55%) | 1.04 | .67–1.61 | .86 |
|  | 75–84 | 311/529 (59%) | 1.23 | .91–1.66 | .18 |
|  | 85–94 | 536/972 (55%) | 1.06 | .80–1.40 | .69 |
|  | ≥ 95 | 137/255 (54%) | Ref | | |
| Death rattles | 65–74 | 61/122 (50%) | 1.00 | .65–1.54 | 1.00 |
|  | 75–84 | 294/560 (53%) | 1.11 | .82–1.49 | .51 |
|  | 85–94 | 521/1014 (51%) | 1.06 | .80–1.39 | .69 |
|  | ≥ 95 | 129/258 (50%) | Ref | | |
| Confusion | 65–74 | 24/108 (22%) | .79 | .46–1.36 | .40 |
|  | 75–84 | 115/503 (23%) | .82 | .57–1.18 | .28 |
|  | 85–94 | 234/904 (26%) | 0.97 | .69–1.35 | .84 |
|  | ≥ 95 | 60/226 (27%) | Ref | | |

**Appendix 2.** Age differences in symptom occurrence in the COVID-19 group, not adjusted for sex. Analysed with logistic regression.

|  | Dementia | No dementia | p value |
| --- | --- | --- | --- |
| Dyspnoea | 244/931 (26%) | 339/961 (35%) | < .001 |
| Pain | 652/948 (69%) | 698/990 (71%) | .41 |
| Nausea | 61/899 (7%) | 115/932 (12%) | < .001 |
| Anxiety | 506/925 (55%) | 542/948 (57%) | .28 |
| Death rattles | 497/958 (52%) | 508/996 (51%) | .70 |
| Confusion | 215/855 (25%) | 218/886 (25%) | .79 |

**Appendix 3.** Differences in symptom occurrence between the groups with and without dementia as a contributing cause of death within the COVID-19 group, not adjusted for age or sex. Analysed with chi2-test.

|  | Women | Men | p value |
| --- | --- | --- | --- |
| Dyspnoea | 150/295 (51%) | 123/288 (43%) | .049 |
| Pain | 641/766 (84%) | 480/584 (82%) | .47 |
| Nausea | 62/120 (52%) | 26/56 (46%) | .52 |
| Anxiety | 454/591 (77%) | 341/457 (75%) | .41 |
| Death rattles | 313/531 (59%) | 232/474 (49%) | .001 |
| Confusion | 87/241 (36%) | 63/192 (33%) | .48 |

**Appendix 4.** Sex differences in complete symptom relief (complete vs partial or not at all) in the COVID-19 group for those reported to have suffered from that symptom during the last week, not adjusted for age. Analysed with chi2-test.

|  | Age group | Complete symptom relief | OR | 95% CI | p value |
| --- | --- | --- | --- | --- | --- |
| Dyspnoea | 65–74 | 18/35 (51%) | 1.41 | .63–3.19 | .41 |
|  | 75–84 | 87/194 (45%) | 1.08 | .63–1.88 | .77 |
|  | 85–94 | 138/284 (49%) | 1.26 | .74–2.14 | .39 |
|  | ≥ 95 | 30/70 (43%) | Ref | | |
| Pain | 65–74 | 74/89 (83%) | .77 | .38–1.56 | .47 |
|  | 75–84 | 318/394 (81%) | .66 | .40–1.08 | .10 |
|  | 85–94 | 576/690 (84%) | .79 | .49–1.27 | .34 |
|  | ≥ 95 | 153/177 (86%) | Ref | | |
| Nausea | 65–74 | 3/8 (38%) | .38 | .07–2.13 | .27 |
|  | 75–84 | 21/54 (39%) | .41 | .14–1.21 | .11 |
|  | 85–94 | 53/96 (55%) | .78 | .28–2.20 | .64 |
|  | ≥ 95 | 11/18 (61%) | Ref | | |
| Anxiety | 65–74 | 45/64 (70%) | .69 | .36–1.35 | .28 |
|  | 75–84 | 237/311 (76%) | .94 | .58–1.51 | .79 |
|  | 85–94 | 407/536 (76%) | .92 | .59–1.44 | .72 |
|  | ≥ 95 | 106/137 (77%) | Ref | | |
| Death rattles | 65–74 | 33/61 (54%) | .99 | .54–1.83 | .98 |
|  | 75–84 | 154/294 (52%) | .93 | .61–1.40 | .72 |
|  | 85–94 | 288/521 (55%) | 1.04 | .71–1.53 | .84 |
|  | ≥ 95 | 70/129 (54%) | Ref | | |
| Confusion | 65–74 | 12/24 (50%) | 1.61 | .62–4.18 | .33 |
|  | 75–84 | 33/115 (29%) | .65 | .34–1.25 | .20 |
|  | 85–94 | 82/234 (35%) | .87 | .48–1.56 | .64 |
|  | ≥ 95 | 23/60 (38%) | Ref | | |

**Appendix 5**. Age differences in complete symptom relief (complete vs partial or not at all) in the COVID-19 group for those reported to have suffered from that symptom during the last week of life, not adjusted for sex. Analysed with logistic regression.
